# Stock price crash risk and military connected board: Evidence from Thailand

**DOI:** 10.1371/journal.pone.0281712

**Published:** 2023-06-01

**Authors:** Sirimon Treepongkaruna, Pattanaporn Chatjuthamard, Arnat Leemakdej

**Affiliations:** 1 Sustainability in Finance & Capital Market Development Research Unit, Sasin School of Management, Chulalongkorn University, Bangkok, Thailand; 2 UWA Business School, The University of Western Australia, Perth, Australia; 3 Center of Excellence in Management Research For Corporate Governance and Behavioral Finance, Sasin School of Management, Chulalongkorn University, Bangkok, Thailand; 4 Thammasat University, Bangkok, Thailand; Massey University - Albany Campus: Massey University - Auckland Campus, NEW ZEALAND

## Abstract

Based on the agency and stakeholder theories, effective boards, acting as an internal governance mechanism, reduce agency costs. This paper asks whether military connected boards represent a good governance tool by exploring how military connected boards affect stock price crash risk. Using instrumental variable analysis, we document that firms with military connected boards have lower risk of stock price crashes. Our findings are unlikely to have endogeneity concerns and shed light on the role of military connected boards as an effective internal governance tool. Consistent with the conservatism hypothesis and stakeholder theory, by being transparent about firm-specific bad news, military connected boards could effectively monitor managers to ensure they act on all stakeholders’ interests.

## 1. Introduction

It is well-documented that stock prices are more susceptible to significant negative movements than extreme positive ones [1–3]. Crash risk, or the occurrence of significant negative returns, jeopardizes firm value, undermines financial market stability, and represents a major concern for shareholders and regulators. In spite of the recent increase in research into the causes of stock price crashes, there is still relatively little evidence on the influence of board characteristics on the probability of crash risk ([3]). We address this void in the literature by examining the role of military directors in determining stock price crash risk, using a unique sample from Thailand, where the military exercises extensive influence. In light of the military’s considerable involvement in Thailand over the past few decades, this country is an especially useful place to study military directors.

Within a company, the board of directors is often considered the ultimate corporate governance instrument. Unsurprisingly, a great deal of research has been conducted on boards of directors. A significant body of research examine the effects of military service on board members and corporate outcomes [4–12]. However, existing literature on military connected boards in developing countries is relatively rare and mainly focus on China, Indonesia and Thailand. For instance, the effect of military connected Chinese boards on post-IPO performance is explored by Fan, Wong and Zhang (2007) [4], and on environmental information disclosures by Cheng, Wang, Kueng and Bai (2017) [6]. Habib and Muhammadi (2018) [8], Nasih, Harymawan, and Putra (2019) [9] and Harymawan (2020) [10] examine military connected boards in Indonesia, and Suriyapongpraprai et al. (2021) [7] and Arayakarnkul, Chatjuthamard and Treepongkaruna (2022) [11] explore the Thai market. While Suriyapongpraprai et al. (2021) [7] focus on military connected boards and firm performance, Arayakarnkul et al. (2022) [11] explore remunerations of military connected board members and top managers. Jaroenjitrkam and Maneenop (2022) [12] also document that Thai firms with military officials on their boards have greater risk, lower accounting performance, but higher market performance than non-military connected firms do. We aptly extend this area of research by focusing on how military directors may impact stock price crash risk in Thailand. Our paper emphasizes on stock price crash risk based on the third moment of the return distribution, measuring the asymmetry of return distribution. This differentiates our research from Jaroenjitrkam and Maneenop (2022) [12], who examine total and idiosyncratic risk, which is the second moment of the return distribution, measuring the spread of distribution and capturing different aspects of risk. To be more specific, the total or idiosyncratic risk is composed of both good and bad news while our stock price crash risk is designed to capture managerial bad news hoarding [13–15].

When compared with China and Indonesia, the Thai political system is unique as its head of state is the monarchy rather than the prime minister. However, since 1932, Thailand has been a constitutional monarchy, where the prime minister is the head of government and a hereditary monarch is the head of state. The 1932 coup, the first coup in Thai history ended absolute monarchy. Since then, Thailand has seen a string of coups, totalling 21 to date. Although Thailand considers itself a democratic country, it is undeniable that the military plays a significant role in directing the country, including its economic policies. The most recent coup in 2014 resulted in a new constitution in 2017, whereby the military continues to exercise power through appointed senators and an array of military-dominated oversight bodies. The influence of military personnel extends to the board of directors of many listed companies. With this unique political system, it is therefore interesting to explore the effect of military connected boards on stock price crash risk in Thailand.

Theoretically, crash risk is predicated on the idea that managers prefer to keep negative news under wraps for long periods of time, enabling it to build up. If managers are effective in preventing negative information from entering the stock market, the distribution of stock returns will be distorted [15,16]. When unfavourable news reaches a certain level, it is disclosed to the market all at once, resulting in a large negative decline in stock price.

Using a sample of large firms in Thailand, we find that more military directors on the board reduce stock price crash risk significantly. Our findings are consistent with those in prior research. Military experience is linked to more conservative corporate practices, according to Benmelech and Frydman (2015) [5]. Military expertise, for instance, results in reduced capital expenditures, less R&D investments, and less debt. Research suggests that military service encourages prudence and hence lowers risk-taking. Managers who are aware of the potential consequences of negative news hoarding are inclined to avoid it, thereby mitigating stock price crash risk. Moreover, our results corroborate the findings in Suriyapongprapai, Chatjuthamard, Suriyapongprapai and Treepongkaruna (2021) [7], who document that military directors in Thai firms significantly raise firm value as measured by Tobin’s q. Overall, our findings support the stakeholders and resource dependency hypotheses, namely that military connected board members improve board oversight and exercise authority to ensure managers act on stakeholders’ interests by not withholding negative news. Our instrumental variable (IV) analyses corroborate main findings and indicate that our results are unlikely to be exposed to endogeneity concerns.

Our research extends the literature in several important ways. First and foremost, our research contributes to an important field of research that examines the impact of military experience on corporate policies and outcomes [5,7,17–19]. Stock price crash risk is influenced significantly by the presence of military connected directors, according to our findings. Second, we make a contribution to the literature on stock price crash risk. Research in this field is critical since a stock market crash might have a severe impact on shareholder welfare [3,13–15]. As far as we know, our analysis is the first to show that military experience on the board is a major predictor of stock price crash risk.

In addition, our study contributes to the body of knowledge on the military’s role in developing countries [7,11,17–19]. The military’s strong position in Thailand makes it an ideal place to study this topic. Our findings suggest that the Thai military has sway over the country’s economy as well as politics, as military board members have a direct impact on important corporate outcomes, such as stock price crash risk.

The remainder of this paper is organised as follows. Section 2 reviews related literature and develops hypotheses. Section 3 describes data and method. Section 4 presents findings and section 5 concludes.

## 2. Pertinent research and hypothesis development

### a. Stock price crash risk

Previous research on crash risk implies that conflicts of interests between controlling insiders and outside investors are a major cause of managerial negative news hoarding and eventual stock price crashes [13–15,20]. Insiders are motivated by agency conflicts to expropriate resources and suppress bad news from the public, which leads to significant overvaluation of company stock [16,21]. Bad news hoarding continues until insiders are no longer able to conceal it. The accumulated negative news is then revealed suddenly, culminating in a stock price crash [3].

Additionally, Bleck and Liu (2007) [14] suggest that managers have an incentive to keep a failed project alive in order to enjoy the benefits of its potential upside. When investors lack the ability to assess the quality of projects, managers conceal substandard performance until negative cash flows arise, precipitating asset price collapse [3]. Such crashes, which may have a tangible impact on investors’ welfare, have grown increasingly prevalent in the aftermath of the financial crisis [3,22].

Anecdotal as well as scholarly evidence support the relationship between agency conflicts and crash risk. Several studies have identified significant correlations between crash risk and corporate governance, financial reporting quality [23], internal control environment [6], and management style [24], all of which are linked to agency costs [3].

Because the board of directors is widely acknowledged as the ultimate instrument of internal governance, board characteristics are crucially important. For instance, Jebran, Chen, and Zhang (2020) [25] document that increasing board diversity can help mitigate the likelihood of a future stock crash risk. Additional findings indicate that board diversity has a great influence on future crash risk for businesses with a high degree of information opacity and low institutional ownership. Wattanatorn and Padungsaksawasdi (2021) [26] created an index for board effectiveness using various attributes, showing that stock price crash risk is significantly related to board attributes.

### b. Military board members

There are advantages to having military connected directors on a company’s board of directors. Self-sacrifice and responsibility are values instilled into those who serve in the military, which may encourage military directors to make ethical judgments and work towards the welfare of the company’s stakeholders, rather than prioritising self-interest [5]. On the other hand, military experience has also been shown to increase aggressiveness, overconfidence, and risk-taking in many individuals [27–29]. Military connected boards are also paid higher, supporting higher agency costs [11].

Military directors may play a distinctive and important function in Asia. As Ghoshal (1986) [30] points out, the military has become a crucial political class and a significant political participant in the late twentieth century. Southeast Asia is a case in point [7]. Although studies on the impact of military ties on companies are rare, there are a few notable instances. Thai and Pakistani stock market returns are much higher during military control than under civilian rule, according to Civilize et al. (2015) [17]. Both Lumjiak et al. (2014) [18] and Lumjiak et al. (2018) [19] indicate that the military coup had a positive effect on the Thai financial market. Having links to the military has been shown to increase corporate performance, according to Suriyapongpraprai et al. (2021) [7]. The findings of Lin et al. (2011) [31] reveal that companies with CEOs with military experience had higher abnormal returns during deal announcement, better synergies, and superior corporate governance. They assert that CEOs with military backgrounds have better judgment and make better decisions [7].

The stakeholder theory and the resource–based theory both suggest that the participation of military board members is advantageous to companies. Directors, according to the resource dependence view, are critical resources for the firm [31,32]. As the number of board members increase, the board’s ability to monitor and advise expands accordingly. Military connected directors contribute distinct and valuable viewpoints, which enhances the board’s efficacy. Since military board members have unique perspectives, they can better connect with the organization’s many stakeholders, according to the stakeholder theory [33]. Arayakarnkul et al. (2022) [11] also find military boards play an effective monitoring role in setting top management pay, supporting the stewardship and stakeholder theories. To sum up, many theories support that having military directors on the board is desirable. The effectiveness of military directors has been shown empirically to improve a company’s overall success.

For decades, the Thai military has played a significant role in its politics. Within the past 15 years, there have been two coups in Thailand. Both coups have resulted in some form of military affiliated government. Since then, there has been a significant rise in the number of military officers serving as board members or chairmen of corporations. Anecdotal evidence suggests that doing business in Thailand requires a high degree of political involvement. The current context of Thai politics has prompted us to investigate if and how military ties impact the degree of stock price crash risk.

### c. Hypothesis development

Based on the literature, two competing hypotheses can be proposed, namely the *conservatism* vs. the *overconfidence* hypotheses. The first hypothesis, the *conservatism hypothesis* states that military directors bring about lower stock price crash risk. According to Benmelech and Frydman (2015) [5], military service is associated with more conservative corporate practices. Military experience, in particular, leads to lower capital expenditures, research and development investment, and leverage. These findings suggest that military service promotes caution and so reduces risk-taking. Prudent managers are probably less likely to practice bad news hoarding as the ultimate outcome could be serious. Therefore, this view predicts that the presence of military directors on the board will result in lower stock price crash risk.

On the other hand, the *overconfidence hypothesis* views that military directors exacerbate stock price crash risk. Military experience is associated with more self-confidence and greater risk-taking, according to psychology research of post-military behavior [5,27–29]. In support of this argument, Padungsaksawasdi, Jiraporn and Treepongkaruna (2022) [34], using a sample of Thai firms, document a higher degree of companies’ risk-taking when there are more military directors on the board, as measured by volatility of earnings while Jaroenjitrkam and Maneenop (2022) [12] find firms with military officials on their boards have greater risk as measured by total or idiosyncratic risk. With greater confidence, military connected directors may see no harm in hoarding bad news. As such, under this hypothesis, having military connected board members lead to higher stock price crash risk.

## 3. Data and modelling framework

### a. Data

We retrieve stock price and firm-specific attributes from Refinitiv and board characteristics from SETSMART. The title of each board members is directly downloaded from SETSMART. When a board member has any of the following titles: Lieutenant General, Vice Admiral, Air Marshal or Police Lieutenant General in the Royal Thai Army, Royal Thai Navy, Royal Thai Air Force, and Royal Thai Police, we classify that person as a military connected director. Data cover firms listed in the SET100 Index during the year 1999 to 2017. To empirically test how firms with military directors serving on boards affect stock price crash risk, we rely on panel regression and two stage least square instrumental variable (IV) analysis with the following model specification:

$${Crash}_{i,T}=\beta_{0}+\beta_{1}{MCON}_{i,T}+\sum \beta_{j}{controls}_{i,T}+\epsilon_{it}$$ (1)

where ***Crash***_***i*,*T***_ represent firm *i* crash risk in year *T* and ***MCOM***_***i*,*T***_ is the proportion of military connected directors on board for firm *i* in year *T*. Two instrumental variable options used in IV analysis are i) the military connections in the earliest year and ii) the industry median of military connections, respectively.

### b. Construction of dependent variable

Our dependent variable is firm-specific crash risk. Two popular proxies of firm-specific stock price crash risk used in existing literature are (i) the negative coefficient of skewness of the firm’s weekly returns (NCSKEW) and (ii) the down-to-up volatility of firm-specific weekly returns (DUVOL). We follow Chen et al (2001) [35] and Hong et al. (2017) [36] to construct these two proxies by first regressing firm’s weekly returns on the current week, two weeks forward, and two weeks backward returns on the MSCI market index as follows:

$$r_{i,t}=\alpha_{i}+\beta_{1,i}r_{m,t}+\beta_{2,i}r_{m,t-1}+\beta_{3,i}r_{m,t-2}+\beta_{4,i}r_{m,t+1}+\beta_{5,i}r_{m,t+2}+\varepsilon_{i,\tau }$$ (2)

where *r*_*i*,*t*_ is the return on stock *i* in week *t* and *r*_*m*,*t*_ is the return on MSCI market index in week *t*. The firm-week return (*W*_*i*,*t*_) is measured as the natural logarithm of one plus the residual return in Eq (1), defined as follows:

$$W_{i,t}=ln(1+\varepsilon_{i,t})$$ (3)

The two proxies of firm-specific stock price crashes can then be defined as follows:

$${NCSKEW}_{i,T}=-[{n(n-1)}^{3/2}\sum W_{i,t}^{3}]/[(n-1)(n-2){\left(\sum W_{i,t}^{2}\right)}^{3/2}]$$ (4)

and

$${DUVOL}_{i,T}=log\{\left[(n_{u}-1)\sum_{DOWN}W_{i,t}^{2}]/[(n_{d}-1)\sum_{UP}W_{i,t}^{2}\right]\}$$ (5)

where *n* represents the number of firm-specific weekly return observations in year *T* and *n*_*u*_ (*n*_*d*_) is the number of weeks with positive (negative) returns in year *T*. As noted in Hong et al. (2017) [36], for each firm in each fiscal year, it is necessary to have at least 20 weeks (or 20 observations) of firm-specific weekly returns. A higher value of NCSKEW or DUVOL indicates a higher crash risk.

### c. Construction of control variables

Existing literature on stock price crash risk identify various variables that can affect crash risk [23,37–39]. These include CEO duality (*DUAL*_*i*,*T*_), the percentage of female directors on board (*Female*_*i*,*T*_), accrual manipulation (*ACCM*_*i*,*T*_) and one-year lag of the following variables: stock price crash risk (*NCSKEW*_*i*_,_*T-1*_), change in stock turnover (*DTURNi*,_*T-1*_), lagged return (*RETi*,_*T-1*_), return volatility (*SIGMAi*,_*T-1*_), firm size (*SIZE*_*i*_,_*T-1*_), book to market (*BMi*,_*T-1*_), leverage (*LEVi*,_*T-1*_) and return on assets (*ROAi*, _*T-1*_). CEO duality (*DUAL*_*i*,*T*_) is a dummy variable indicating 1 if the CEO for firm *i* in year *T* concurrently serves as the board chairperson and zero otherwise. Accrual manipulation (*ACCM*_*i*,*T*_) is calculated as the three-year moving summation of absolute discretionary accruals for firm *i*. We control for this variable as it was found to be a significant determinant of crash risk. Hutton, Marcus, and Tehranian (2009) [15] document a positive relation between ACCM and crash risk, implying that the opacity of a firm’s financial statements allow managers to obscure negative information about underlying fundamentals. However, it was argued that a financial statement’s bottom line is not the only way to convey information. Managers may withhold bad news from investors using tools other than manipulating a firm’s accrual [38,40]. Additionally, the firm-level control variables are chosen based on existing literature that considers what explains firm-specific crash risk. Following Chen, Hong, and Stein (2001) [33], Hutton, Marcus, and Tehranian (2009) [15], Kim, Li, and Zhang (2011a,b) [37,38], and Hong, Kim and Welker (2017) [36], we include one-year lagged measures of firm-level control variables. The change in stock turnover (*DTURNi*,_*T-1*_) is computed as the difference between average monthly stock turnover in the fiscal year *T-1* and *T-2*. Lagged return (*RETi*,_*T-1*_) is the average firm-specific weekly returns over the fiscal year *T-1*; while lagged volatility (*SIGMAi*,_*T-1*_) is the standard deviation of firm-specific weekly returns in the fiscal year *T-1*. The remaining accounting control variables are defined as follows: lagged firm size (*SIZE*_*i*,*T-1*_) is defined as the natural logarithm of firm *i* market value in the fiscal year *T-1*; *BM*_*i*,*T-1*_ is defined as the book-to-market ratio for firm *i* in the fiscal year *T-1*; *LEV*_*i*,*T-1*_ is defined as the ratio of long-term debt over total assets for firm *i* in the fiscal year *T-1*; and *ROA*_*i*,*T-1*_ is defined as the ratio of net income over total assets for firm *i* in the fiscal year *T-1*.

### d. Summary statistics

Table 1 reports the summary statistics for all the variables used in this study. The mean values of our crash risk measures, NCSKEW_i,T_ and DUVOL_i,T_ are -0.13 and -0.10, respectively. The average percentage of military connected board members is 3% while the average CEO duality and percentage of female directors is slightly higher at around 10–11%. With respect to our control variables, the mean *DTURN*_*i*,*T-1*_ is -0.12, the average *RET*_*i*,*T-1*_ is 0.004, and the average *SIGMA*_*i*,*T-1*_ is 0.05. Consistent with existing literature, the two proxies of crash risk are highly correlated. In addition, we find negative correlation between *MCON*_*i*,*T*_ and *Female*_*i*,*T*_, and *MCON*_*i*,*T*_ and *Dual*_*i*,*T*_. It appears firms with military connected board members do not appoint the CEO as the chairperson of the board, and tend to have lower percentages of female directors. This implies military experience substitute the role of female directors in strengthening good governance.

**Table 1:** a: Descriptive Statistics. b: Correlation Matrix.

Variable	Obs	Mean	Std. Dev.	Min	Max	p5	p95
*NCSKEW*_*i*,*T*_	703	-0.131714	0.672377	-2.694638	2.773906	-1.210421	0.970779
*DUVOL*_*i*,*T*_	703	-0.104231	0.486166	-1.53454	1.614186	-0.884946	0.731868
*MCON*_*i*,*T*_	703	0.031378	0.057902	0	0.4	0	0.166667
*NCSKEW*_*i*,*T-1*_	703	-0.000152	0.073018	-0.339927	0.408451	-0.097706	0.098045
*DTURN*_*i*,*T-1*_	703	-0.124714	0.675111	-2.610891	2.701039	-1.210627	0.977411
*RET*_*i*,*T-1*_	703	0.003655	0.009758	-0.022217	0.033914	-0.010193	0.02073
*SIGMA*_*i*,*T-1*_	703	0.051242	0.022764	0.014068	0.19661	0.02685	0.094271
*SIZE*_*i*,*T-1*_	703	21.10255	1.639135	18.24938	25.1518	18.78413	24.38862
*MB*_*i*,*T-1*_	703	2.516164	2.380683	0.202354	14.37633	0.59688	7.067755
*LEV*_*i*,*T-1*_	703	0.334233	0.194486	0	0.720008	0.003386	0.697145
*ROA*_*i*,*T-1*_	703	8.578516	7.061739	-14.21401	28.98438	0.58583	21.46008
*ACCM*_*tI*,*T1*_	703	0.153135	0.167391	1.39E-17	1.21779	0.006936	0.482444
*Female*_*i*,*T*_	703	0.11612	0.101117	0	0.5	0	0.3
*Dual*_*i*,*T*_	703	0.109531	0.312526	0	1	0	1

		1	2	3	4	5	6	7	8	9	10	11	12	13	14
1	*NCSKEW*_*i*,*T*_	1													
2	*DUVOL*_*i*,*T*_	0.8849	1												
3	*MCON*_*i*,*T*_	-0.0581	-0.0511	1											
4	*NCSKEW*_*i*,*T-1*_	-0.0311	-0.0133	0.0035	1										
5	*DTURN*_*i*,*T-1*_	-0.0214	-0.0309	-0.032	-0.0519	1									
6	*RET*_*i*,*T-1*_	0.1054	0.1159	-0.0201	0.3665	-0.2909	1								
7	*SIGMA*_*i*,*T-1*_	-0.0218	-0.0157	0.0629	0.307	-0.0125	0.1949	1							
8	*SIZE*_*i*,*T-1*_	0.0242	0.0298	0.2831	-0.0184	0.0214	-0.0994	-0.2061	1						
9	*MB*_*i*,*T-1*_	0.1481	0.1701	-0.0832	0.0764	0.0164	0.1825	-0.0475	-0.0183	1					
10	*LEV*_*i*,*T-1*_	-0.0773	-0.0804	0.0797	0.0138	-0.0874	0.0086	0.1757	-0.2231	-0.1196	1				
11	*ROA*_*i*,*T-1*_	0.1109	0.1109	-0.0865	0.1131	0.0718	0.2098	-0.0877	-0.3212	0.462	-0.2542	1			
12	*ACCM*_*tI*,*T1*_	0.0967	0.1008	-0.1457	0.0225	0.0617	-0.0438	-0.0051	-0.2495	0.4205	-0.2331	0.5857	1		
13	*Female*_*i*,*T*_	0.0567	0.0464	-0.2125	-0.0026	0.0865	-0.0039	-0.0332	-0.1965	0.1067	-0.123	0.0977	0.1906	1	
14	*Dual*_*i*,*T*_	-0.072	-0.0476	-0.1902	-0.017	-0.0698	0.026	0.0003	-0.207	0.1003	0.0011	0.1296	0.178	0.2463	1

The table presents descriptive statistics for all variables included in this paper. Sample are from 1999 to 2017. *NCSKEW*_*i*,*T*_, is the stock price crash risk for firm *i* at time *T*. *DUVOL*_*i*,*T*_ is the down-to-up volatility for firm *i* at time *T*. *DTURN*_*i*,*T*_, is the change in stock turnover ratio (calculated as the difference between the average monthly stock turnover for firm *i* in the fiscal year T-1 and the average monthly turnover in the fiscal year T-2). *RET*_*i*,*T*_, is the average of firm-specific weekly returns over the fiscal year T-1. *SIGMA*_*i*,*T-1*_ is the standard deviation of the firm-specific weekly returns in the fiscal year T-1. *SIZE*_*i*,*T-1*_, is the natural logarithm of firm *i* market value in the fiscal year T-1. *MB*_*tI*,*T1*_is the market-to-book ratio for firm *i* in the fiscal year T-1. *LEV*_*i*,*T-1*_ is the ratio of long-term debt over total assets for firm *i* in the fiscal year T-1. *ROA*_*i*,*T-1*_ is the ratio of net income over total assets for firm *i* in the fiscal year T-1. Accrual manipulation (*ACCM*_*i*,*T*_) is the three-year moving summation of absolute discretionary accruals. *MCON*_*i*,T_ represents percentage of directors with military connection on board for firm *i* in year *T*. *Female*_i,T_ stands for percentages of female director on board for firm *i* in year *T*. *Dual*_i,T_ is a dummy variable indicating 1 if CEO concurrently serves as the board chairperson and zero otherwise for firm *i* in year *T*.

## 4. Results

Table 2 provide results from the ordinary least squares regression with clustered standard errors of model 1, where our dependent variables are both proxies of crash risk (*NCSKEW*_*i*,*T*_ and *DUVOL*_*i*,*T*_). Consistent with our *conservatism hypothesis*, we find negative associations between *MCON*_*i*,*T*_ and both proxies of crash risk. Table 2 indicates the coefficient of the proportion of military directors (*MCON*_*i*,*T*_*)* in Models 1 and 2 are -0.95 and -0.6, respectively. As the standard deviation of the proportion of military directors is 0.058, these coefficients imply that an increase in one standard deviation of the percentage of military board members lead to a decline in stock price crash risk by 7–8%. That is, for Model 1, with one standard deviation increase in MCON_*i*,*T*_, the crash risk declines by 0.058 times -0.95, which is equivalent to -0.055. As the standard deviation of *NCSKEW*_*i*,*T*_ is 0.67, a drop by 0.055 equates to 8%. Similarly, the coefficient of -0.6 in Model 2 implies a 7% reduction in crash risk. Our findings are both statistically and economically significant.

**Table 2:** Effect of military connected directors on stock price crash risk.

	*NCSKEW*_*i*,*T*_	*DUVOL*_*i*,*T*_
*MCON*_*i*,*T*_	-0.945**	-0.602*
	(0.463)	(0.330)
*DTURN*_*i*,*T-1*_	-0.846**	-0.433
	(0.396)	(0.282)
*NCSKEW*_*i*,*T-1*_	0.00510	0.00216
	(0.0394)	(0.0280)
*RET*_*i*,*T-1*_	7.674*	5.264*
	(3.969)	(2.824)
*SIGMA*_*i*,*T-1*_	1.281	0.561
	(1.594)	(1.134)
*SIZE*_*i*,*T-1*_	0.0383*	0.0297**
	(0.0198)	(0.0141)
*MB*_*i*,*T-1*_	0.0215*	0.0213**
	(0.0127)	(0.00901)
*LEV*_*I*,*T1*_	-0.0487	-0.0502
	(0.145)	(0.103)
*ROA*_*i*,*T-1*_	0.00684	0.00370
	(0.00521)	(0.00371)
*ACCM*_*i*,*T*_	0.158	0.119
	(0.199)	(0.142)
*Female*_*i*,*T*_	0.428	0.233
	(0.260)	(0.185)
*Dual*_*i*,*T*_	-0.240***	-0.121**
	(0.0830)	(0.0591)
Constant	-1.281**	-0.927**
	(0.527)	(0.375)
Observations	703	703
R-squared	0.127	0.155

This table reports regression results where our main dependent variable is *CRASH_RISK*_*i*,*T*_ represents the stock price crash measures: *NCSKEW*_*i*,*T*_, or *DUVOL*_*i*,*T*_. Our key variable of interest, *MCON*_i,T_, represents percentage of directors with military connection on board for firm *i* in year *T*. Definition of control variables are in S1 Table. Sample period spans from 1999 to 2017 for firms in SET100 index. ***,**,* indicates significant level of 1%,5% and 10%, respectively. Clustered standard errors includes in the parentheses.

Tables 3 and 4 report results from the two-stage least squares instrumental variable analyses, where the dependent variables are *NCKEW*_*i*,*T*_ and *DUVOL*_*i*,*T*_, respectively. We conduct the two-stage least square instrumental variable analysis to ensure our findings are robust and less likely to have endogeneity concerns. In doing so, we employ two instrumental variables, popularly used in existing literature. The first instrumental variable is the percentage of military directors in the earliest year for each firm, while the second is the industry median of the percentage of military directors. These two instrumental variables appear to meet both relevance and exclusion restrictions. That is, both instruments are highly correlated with the endogenous explanatory variables at the 1% statistical significance level, conditional on the other covariates. Both instruments are likely to meet exclusion restrictions, as the value of *MCON* in the earliest year is unlikely to have any impact on the stock price crash risk in a subsequent year. Similarly, the presence of military board members at the industry level is less likely to affect a firm’s stock price crash risk. Further, to ensure our results are not driven by macroeconomic factors, we split our sample into two sub samples representing different periods: global financial crisis (GFC) period vs. non-GFC period and report findings in S2 and S3 Tables. Results from Tables 3, 4, S2 and S3 confirm our main findings in Table 2 that having more military connected board members reduce stock price crash risk, consistent with *conservatism hypothesis*. S2 and S3 Tables also indicate that our results are not driven by macroeconomic factors. Based on the IV approach, our findings are unlikely exposed to endogeneity and indicate a causal effect (not merely an association) of military connected board members on stock price crash.

**Table 3:** Causal relation between military connected directors and stock price crash risk *(NCSKEW)*.

	*NCSKEW*_*i*,*T*_			
	1st Stage	2nd stage	1st Stage	2nd stage
MCON_i,T_		-3.355**		-1.754**
		(1.398)		(0.748)
*DTURN*_*i*,*T-1*_	0.00404	-0.782**	0.00317	-0.865**
	(0.0239)	(0.390)	(0.0262)	(0.388)
*NCSKEW*_*i*,*T-1*_	0.000500	-0.0449	0.000482	0.00256
	(0.00239)	(0.0388)	(0.00261)	(0.0386)
*RET*_*i*,*T-1*_	-0.210	5.931	-0.288	7.258*
	(0.241)	(3.956)	(0.263)	(3.901)
*SIGMA*_*i*,*T-1*_	0.0590	1.534	0.224**	1.677
	(0.106)	(1.731)	(0.105)	(1.588)
*SIZE*_*i*,*T-1*_	0.00785***	0.0475	0.00648***	0.0488**
	(0.00204)	(0.0345)	(0.00129)	(0.0209)
*MB*_*i*,*T-1*_	-0.000378	0.0384**	-0.00106	0.0200
	(0.00102)	(0.0166)	(0.000838)	(0.0125)
*LEV*_*I*,*T1*_	-0.00936	-0.0477	-0.00509	-0.0143
	(0.0130)	(0.212)	(0.00979)	(0.144)
*ROA*_*i*,*T-1*_	0.000834**	0.00876	0.000325	0.00812
	(0.000344)	(0.00565)	(0.000348)	(0.00519)
*ACCM*_*i*,*T*_	-0.00653	-0.243	-0.00910	0.138
	(0.0152)	(0.248)	(0.0132)	(0.196)
*Female*_*i*,*T*_	-0.00362	0.607**	-0.0150	0.387
	(0.0181)	(0.295)	(0.0173)	(0.257)
*Dual*_*i*,*T*_	-0.0106*	-0.380***	-0.00998*	-0.254***
	(0.00607)	(0.101)	(0.00549)	(0.0821)
EARLIEST_MCON	0.259***			
	(0.0223)			
MCON_MED			0.727***	
			(0.0368)	
Constant	-0.157***	-1.556*	-0.130***	-1.528***
	(0.0476)	(0.803)	(0.0348)	(0.548)
Year FE	Yes	Yes	Yes	Yes
Industry FE	Yes	Yes		
Observations	703	703	703	703
R-squared	0.603	0.153	0.484	0.123

This table reports 2SLS IV results where our main dependent variable is *CRASH_RISK*_*i*,*T*_ represents the stock price crash measures: *NCSKEW*_*i*,*T*_. Our key variable of interest, *MCON*_i,T_, represents percentage of directors with military connection on board for firm *i* in year *T*. The MCON first appeared in our sample (Earliest_MCON) or the industry median of MCON (MCON_MED) is used as instrument. Definition of control variables are in S1 Table. Sample period spans from 1999 to 2017 for firms in SET100 index.

***,**,* indicates significant level of 1%,5% and 10%, respectively. Clustered standard errors includes in the parentheses.

**Table 4:** Causal relation between military connected directors and stock price crash risk *(DUVOL)*.

	*DUVOL*_*i*,*T*_			
	1st Stage	2nd stage	1st Stage	2nd stage
MCON_i,T_		-1.662*		-1.155**
		(0.988)		(0.532)
*DTURN*_*i*,*T-1*_	0.00404	-0.424	0.00317	-0.445
	(0.0239)	(0.276)	(0.0262)	(0.276)
*NCSKEW*_*i*,*T-1*_	0.000500	-0.0317	0.000482	0.000429
	(0.00239)	(0.0274)	(0.00261)	(0.0275)
*RET*_*i*,*T-1*_	-0.210	4.460	-0.288	4.981*
	(0.241)	(2.798)	(0.263)	(2.775)
*SIGMA*_*i*,*T-1*_	0.0590	0.639	0.224**	0.832
	(0.106)	(1.224)	(0.105)	(1.130)
*SIZE*_*i*,*T-1*_	0.00785***	0.0237	0.00648***	0.0369**
	(0.00204)	(0.0244)	(0.00129)	(0.0149)
*MB*_*i*,*T-1*_	-0.000378	0.0254**	-0.00106	0.0202**
	(0.00102)	(0.0117)	(0.000838)	(0.00887)
*LEV*_*I*,*T1*_	-0.00936	0.0831	-0.00509	-0.0267
	(0.0130)	(0.150)	(0.00979)	(0.102)
*ROA*_*i*,*T-1*_	0.000834**	0.00665*	0.000325	0.00457
	(0.000344)	(0.00399)	(0.000348)	(0.00369)
*ACCM*_*i*,*T*_	-0.00653	-0.0646	-0.00910	0.105
	(0.0152)	(0.176)	(0.0132)	(0.139)
*Female*_*i*,*T*_	-0.00362	0.359*	-0.0150	0.206
	(0.0181)	(0.208)	(0.0173)	(0.183)
*Dual*_*i*,*T*_	-0.0106*	-0.191***	-0.00998*	-0.130**
	(0.00607)	(0.0713)	(0.00549)	(0.0584)
EARLIEST_MCON	0.259***			
	(0.0223)			
MCON_MED			0.727***	
			(0.0368)	
Constant	-0.157***	-0.954*	-0.130***	-1.095***
	(0.0476)	(0.568)	(0.0348)	(0.390)
Year FE	Yes	Yes	Yes	Yes
Industry FE	Yes	Yes		
Observations	703	703	703	703
R-squared	0.603	0.190	0.484	0.151

This table reports 2SLS IV results where our main dependent variable is *CRASH_RISK*_*i*,*T*_ represents the stock price crash measures: *DUVOL*_*i*,*T*._ Our key variable of interest, *MCON*_i,T_, represents percentage of directors with military connection on board for firm *i* in year *T*. The MCON first appeared in our sample (Earliest_MCON) or the industry median of MCON (MCON_MED) is used as instrument. Definition of control variables are in S1 Table. Sample period spans from 1999 to 2017 for firms in SET100 index.

***,**,* indicates significant level of 1%,5% and 10%, respectively. Clustered standard errors includes in the parentheses.

## 5. Conclusions

A significant drop in a company’s adjusted stock return is the definition of a stock price crash [15]. As a result, such sharp declines not only reduce the portfolio’s returns but also raise the portfolio’s level of risk [41]. Crash risk became a concern for investors and regulators following a slew of high-profile corporate scandals in the early 2000s. In addition, the devastating outcomes of the 2008 financial crisis created a significant incentive for further investigation into the causes of stock price crashes [41]. Hoarding bad news leads to stock price crashes. Agency theory often argues that managers have incentives to hoard bad news. A corporate governance tool used in curbing agency costs is the board of directors. A well-chosen board of directors will provide effective monitoring and oversight. Focusing on a specific trait of these directors, being military experience, we test two contrasting hypotheses, namely *conservatism* vs. *overconfidence*. Consistent with the stakeholder theory, our findings provide support for the *conservatism* hypothesis. That is, board members with military experience do not hoard bad news. As such, firms with military connected boards tend to have lower crash risk. It appears military connected board members effectively fulfill the monitoring role, keeping in check managers’ bad news hoarding.

As noted by Benmelech and Frydman (2015) [5], the role of military board members is an important area of research. We contribute to this area of literature by documenting that the presence of board members with military experience is one of the key determinants of stock price crash risk in Thailand, where the military have a strong influence over business culture and play a vital role in many parts of the country. Our findings are consistent with those in Suriyapongpraprai et al. (2021) [7] and Arayakarnkul e al. (2022) [11], who find that board members with military experience are better able to monitor and direct managers to act on all stakeholders’ interests. Further, consistent with Benmelech and Frydman (2015) [5], we find that military experience is associated with more conservative business policies.

As our study only focuses on the top 100 Thai listed firms, we cannot conclude that the effect of having military connected board members on crash risk is the same in other countries with different environments and business cultures. The military is revered in some countries, while others may not hold them in high regard. It is likely that the military as an institution could function differently in different cultures, hence its effect is not necessary the same across countries. Future research could explore our concept in other markets.

## Supporting information

S1 Table A1: Definition of all variablesTable. (DOCX)

S2 Table A2: Causal relation between Military Connected Directors and Stock Price Crash Risk (NCSKEW): Global Financial Crisis (GFC). (DOCX)

S3 Table A3: Causal relation between Military Connected Directors and Stock Price Crash Risk *(DUVOL)*: Global Financial Crisis (GFC). (DOCX)
