# Supplementary material for: Stock price crash risk and military connected board: Evidence from Thailand
Source: PLoS One. 2023 Jun 1;18(6):e0281712. doi: 10.1371/journal.pone.0281712 (PMC10234529; doi:10.1371/journal.pone.0281712)
Supplement: S1 Table — (DOCX) [file pone.0281712.s001.docx]

**Table A1: Definition of all variables**

| **Variables** | **Definition** |
| --- | --- |
| *NCSKEW* | Negative skewness of firm-specific weekly returns over the fiscal year period. |
| *DUVOL* | Down-to-up volatility of firm-specific weekly returns |
| *MCON* | Percentage of directors with military connection on board |
| *DTURN* | Average monthly share turnover over the current fiscal year period minus the average monthly share turnover over the previous fiscal year period, where monthly share turnover is calculated as the monthly trading volume divided by the total number of shares outstanding during the month |
| *RET* | Average of firm-specific weekly returns over the fiscal year |
| *SIGMA* | Standard deviation of the firm-specific weekly returns over the fiscal year |
| *SIZE* | Natural logarithm of firm market value |
| *MB* | Market-to-book ratio |
| *LEV* | Ratio of long-term debt over total assets |
| *ROA* | Ratio of net income over total assets |
| *ACCM* | Three-year moving summation of absolute discretionary accruals |
| *Female* | Percentages of female director on board |
| *Dual* | A dummy variable indicating 1 if CEO concurrently serves as the board chairperson and zero otherwise |
