# Supplementary material for: Stock price crash risk and military connected board: Evidence from Thailand
Source: PLoS One. 2023 Jun 1;18(6):e0281712. doi: 10.1371/journal.pone.0281712 (PMC10234529; doi:10.1371/journal.pone.0281712)
Supplement: S2 Table — (DOCX) [file pone.0281712.s002.docx]

**Table A2:** **Causal relation between Military Connected Directors and Stock Price Crash Risk (NCSKEW): Global Financial Crisis (GFC)**

This table reports 2SLS IV results where our main dependent variable is *CRASH_RISKi,T* represents the stock price crash measures: *NCSKEWi,T*. Our key variable of interest, *MCONi,T*, represents percentage of directors with military connection on board for firm i in year T. The MCON first appeared in our sample *(Earliest_MCON*) or the industry median of MCON *(MCON_MED)* is used as instrument. Definition of control variables are in Table A1 in Appendix. GFC sample period spans from 2007 to 2009 and non-GFC sample is from 1999 to 2006 and 2010 to 2017 for firms in SET100 index. ***,**,* indicates significant level of 1%,5% and 10%, respectively. Clustered standard errors includes in the parentheses.

|  | ***GFC Period*** | | | | ***Non-GFC Period*** | | | |
| --- | --- | --- | --- | --- | --- | --- | --- | --- |
| ***NCSKEW*** | 1st Stage | 2nd Stage | 1st Stage | 2nd Stage | 1st Stage | 2nd Stage | 1st Stage | 2nd Stage |
| MCON |  | -6.359** |  | -3.116 |  | -2.951** |  | -1.336* |
|  |  | (2.757) |  | (2.077) |  | (1.503) |  | (0.797) |
| *DTURN _i,T-1_* | 0.215** | -1.466 | 0.0500 | -2.031 | -0.00641 | -0.852** | -0.000349 | -0.923** |
|  | (0.103) | (1.652) | (0.113) | (1.551) | (0.0250) | (0.406) | (0.0274) | (0.405) |
| *NCSKEW_i,T-1_* | 0.00375 | -0.235*** | 0.00367 | -0.152* | -0.000322 | -0.0319 | -0.000461 | 0.0251 |
|  | (0.00543) | (0.0860) | (0.00580) | (0.0798) | (0.00269) | (0.0437) | (0.00294) | (0.0435) |
| *RET_i,T-1_* | -0.000488 | 5.320 | -0.0849 | 3.533 | -0.312 | 6.644 | -0.312 | 7.435* |
|  | (0.624) | (9.877) | (0.689) | (9.496) | (0.268) | (4.406) | (0.289) | (4.286) |
| *SIGMA _i,T-1_* | -0.330 | -4.710 | 0.428* | -1.389 | 0.122 | 2.979 | 0.207* | 3.149* |
|  | (0.277) | (4.201) | (0.247) | (3.637) | (0.120) | (1.973) | (0.120) | (1.797) |
| *SIZE _i,T-1_* | 0.00761 | 0.0957 | 0.00525* | 0.0591 | 0.00836*** | 0.0467 | 0.00718*** | 0.0487** |
|  | (0.00499) | (0.0805) | (0.00305) | (0.0463) | (0.00228) | (0.0385) | (0.00145) | (0.0235) |
| *MB _i,T-1_* | -0.00592 | 0.175** | -0.00625* | 0.110** | -6.29e-06 | 0.0282 | -0.000772 | 0.0117 |
|  | (0.00457) | (0.0710) | (0.00326) | (0.0483) | (0.00109) | (0.0177) | (0.000880) | (0.0130) |
| *LEV_I,T1_* | -0.0303 | 1.067 | 0.0100 | 0.384 | -0.0108 | -0.218 | -0.00837 | -0.126 |
|  | (0.0577) | (0.922) | (0.0247) | (0.344) | (0.0140) | (0.228) | (0.0109) | (0.159) |
| *ROA_i,T-1_* | 0.00136 | -0.0142 | 0.000394 | -0.0119 | 0.000896** | 0.0109* | 0.000388 | 0.0131** |
|  | (0.000959) | (0.0147) | (0.000809) | (0.0114) | (0.000386) | (0.00634) | (0.000397) | (0.00592) |
| *ACCM_i,T_* | -0.00646 | 0.233 | 0.0432 | 0.625 | -0.00533 | -0.348 | -0.0155 | 0.0758 |
|  | (0.0605) | (0.957) | (0.0405) | (0.558) | (0.0163) | (0.265) | (0.0142) | (0.210) |
| *Female_i,T_* | -0.0235 | 1.105 | -0.0235 | 0.775 | 0.000610 | 0.594* | -0.0107 | 0.329 |
|  | (0.0440) | (0.702) | (0.0419) | (0.585) | (0.0202) | (0.328) | (0.0191) | (0.283) |
| *Dual_i,T_* | -0.0220 | -0.554** | -0.00558 | -0.451** | -0.00881 | -0.329*** | -0.0102* | -0.231*** |
|  | (0.0165) | (0.276) | (0.0148) | (0.204) | (0.00673) | (0.111) | (0.00598) | (0.0894) |
| EARLIEST_MCON | 0.257*** |  |  |  | 0.276*** |  |  |  |
|  | (0.0448) |  |  |  | (0.0256) |  |  |  |
| MCON_industry_med |  |  | 0.580*** |  |  |  | 0.757*** |  |
|  |  |  | (0.0876) |  |  |  | (0.0408) |  |
| Constant | -0.148 | -2.357 | -0.132* | -1.652 | -0.174*** | -1.650* | -0.140*** | -1.673*** |
|  | (0.119) | (1.919) | (0.0746) | (1.134) | (0.0531) | (0.896) | (0.0386) | (0.607) |
| Year FE | Yes | Yes | Yes | Yes | Yes | Yes | Yes | Yes |
| Industry FE | Yes | Yes |  |  | Yes | Yes |  |  |
| Observations | 131 | 131 | 131 | 131 | 572 | 572 | 572 | 572 |
| R-squared | 0.678 | 0.370 | 0.432 | 0.324 | 0.623 | 0.143 | 0.502 | 0.104 |
