# Supplementary material for: Stock price crash risk and military connected board: Evidence from Thailand
Source: PLoS One. 2023 Jun 1;18(6):e0281712. doi: 10.1371/journal.pone.0281712 (PMC10234529; doi:10.1371/journal.pone.0281712)
Supplement: S3 Table — (DOCX) [file pone.0281712.s003.docx]

**Table A3:** **Causal relation between Military Connected Directors and Stock Price Crash Risk *(DUVOL):* Global Financial Crisis (GFC)**

This table reports 2SLS IV results where our main dependent variable is *CRASH_RISKi,T* represents the stock price crash measures: *DUVOLi,t* Our key variable of interest, *MCONi,T*, represents percentage of directors with military connection on board for firm i in year T. The MCON first appeared in our sample *(Earliest_MCON*) or the industry median of MCON *(MCON_MED)* is used as instrument. Definition of control variables are in Table A1 in Appendix. GFC sample period spans from 2007 to 2009 and non-GFC sample is from 1999 to 2006 and 2010 to 2017 for firms in SET100 index. ***,**,* indicates significant level of 1%,5% and 10%, respectively. Clustered standard errors includes in the parentheses.

|  | ***GFC Period*** | | | | ***Non-GFC Period*** | | | |
| --- | --- | --- | --- | --- | --- | --- | --- | --- |
| ***DUVOL*** | 1st Stage | 2nd Stage | 1st Stage | 2nd Stage | 1st Stage | 2nd Stage | 1st Stage | 2nd Stage |
| MCON |  | -4.384** |  | -0.539 |  | -1.252 |  | -1.044* |
|  |  | (2.066) |  | (1.525) |  | (1.045) |  | (0.563) |
| *DTURN _i,T-1_* | 0.215** | 0.310 | 0.0500 | -0.348 | -0.00641 | -0.494* | -0.000349 | -0.528* |
|  | (0.103) | (1.238) | (0.113) | (1.138) | (0.0250) | (0.282) | (0.0274) | (0.286) |
| *NCSKEW_i,T-1_* | 0.00375 | -0.130** | 0.00367 | -0.0964* | -0.000322 | -0.0323 | -0.000461 | 0.0120 |
|  | (0.00543) | (0.0644) | (0.00580) | (0.0585) | (0.00269) | (0.0304) | (0.00294) | (0.0307) |
| *RET_i,T-1_* | -0.000488 | 8.097 | -0.0849 | 4.764 | -0.312 | 4.711 | -0.312 | 4.426 |
|  | (0.624) | (7.400) | (0.689) | (6.970) | (0.268) | (3.064) | (0.289) | (3.028) |
| *SIGMA _i,T-1_* | -0.330 | -3.768 | 0.428* | -2.916 | 0.122 | 1.536 | 0.207* | 2.114* |
|  | (0.277) | (3.147) | (0.247) | (2.670) | (0.120) | (1.372) | (0.120) | (1.269) |
| *SIZE _i,T-1_* | 0.00761 | 0.0943 | 0.00525* | 0.0226 | 0.00836*** | 0.0121 | 0.00718*** | 0.0403** |
|  | (0.00499) | (0.0603) | (0.00305) | (0.0340) | (0.00228) | (0.0268) | (0.00145) | (0.0166) |
| *MB _i,T-1_* | -0.00592 | 0.104* | -0.00625* | 0.103*** | -6.29e-06 | 0.0193 | -0.000772 | 0.0134 |
|  | (0.00457) | (0.0532) | (0.00326) | (0.0355) | (0.00109) | (0.0123) | (0.000880) | (0.00922) |
| *LEV_I,T1_* | -0.0303 | 1.001 | 0.0100 | 0.250 | -0.0108 | -0.0382 | -0.00837 | -0.115 |
|  | (0.0577) | (0.691) | (0.0247) | (0.252) | (0.0140) | (0.158) | (0.0109) | (0.112) |
| *ROA_i,T-1_* | 0.00136 | -0.0133 | 0.000394 | -0.0111 | 0.000896** | 0.00780* | 0.000388 | 0.00848** |
|  | (0.000959) | (0.0110) | (0.000809) | (0.00839) | (0.000386) | (0.00441) | (0.000397) | (0.00418) |
| *ACCM_i,T_* | -0.00646 | 0.321 | 0.0432 | 0.141 | -0.00533 | -0.144 | -0.0155 | 0.0806 |
|  | (0.0605) | (0.717) | (0.0405) | (0.409) | (0.0163) | (0.184) | (0.0142) | (0.148) |
| *Female_i,T_* | -0.0235 | 0.969* | -0.0235 | 0.607 | 0.000610 | 0.294 | -0.0107 | 0.144 |
|  | (0.0440) | (0.526) | (0.0419) | (0.429) | (0.0202) | (0.228) | (0.0191) | (0.200) |
| *Dual_i,T_* | -0.0220 | -0.301 | -0.00558 | -0.281* | -0.00881 | -0.143* | -0.0102* | -0.114* |
|  | (0.0165) | (0.207) | (0.0148) | (0.150) | (0.00673) | (0.0771) | (0.00598) | (0.0631) |
| EARLIEST_MCON | 0.257*** |  |  |  | 0.276*** |  |  |  |
|  | (0.0448) |  |  |  | (0.0256) |  |  |  |
| MCON_industry_med |  |  | 0.580*** |  |  |  | 0.757*** |  |
|  |  |  | (0.0876) |  |  |  | (0.0408) |  |
| Constant | -0.148 | -2.377* | -0.132* | -0.665 | -0.174*** | -0.777 | -0.140*** | -1.282*** |
|  | (0.119) | (1.438) | (0.0746) | (0.832) | (0.0531) | (0.623) | (0.0386) | (0.429) |
| Year FE | Yes | Yes | Yes | Yes | Yes | Yes | Yes | Yes |
| Industry FE | Yes | Yes |  |  | Yes | Yes |  |  |
| Observations | 131 | 131 | 131 | 131 | 572 | 572 | 572 | 572 |
| R-squared | 0.678 | 0.376 | 0.432 | 0.357 | 0.623 | 0.191 | 0.502 | 0.127 |
